# The Relationship between Personal Resources and Depression in a Sample of Victims of Cyberbullying: Comparison of Groups with and without Symptoms of Depression

**DOI:** 10.3390/ijerph17249307

**Published:** 2020-12-12

**Authors:** Lourdes Rey, Cirenia Quintana-Orts, Sergio Mérida-López, Natalio Extremera

**Affiliations:** 1 Department of Personality, Evaluation and Psychological Treatment, University of Malaga, 29071 Malaga, Spain; 2 Department of Personality, Evaluation and Psychological Treatment, University of Granada, 51001 Granada, Spain; cquintana@ugr.es; 3 Department of Social Psychology, Social Work, Social Anthropology and East Asian Studies, University of Malaga, 29071 Malaga, Spain; sergioml@uma.es (S.M.-L.); nextremera@uma.es (N.E.)

**Keywords:** cyberbullying, victimisation, adolescents, depressive symptomatology, personal resources

## Abstract

Previous research has highlighted the relationship between being cybervictimised and the presence of clinical symptoms, such as depression. To date, however, there has been no comparative analysis of the personal resources profiles of adolescent victims of cyberbullying with and without depressive symptoms. The current study analysed the relationship between positive personal resources and clinical symptoms in 251 adolescent victims of cyberbullying at several Spanish high schools. It examined how several positive personal resources varied in adolescent victims of cyberbullying who displayed symptoms of depression (*n =* 89) or did not (*n =* 162). Victims of cyberbullying who displayed depressive symptoms reported lower levels of personal resources (emotional intelligence, gratitude, optimism, and forgiveness) than those who did not. Logistic regression provided evidence that gratitude was the strongest predictor of depressive symptoms in victims of cyberbullying, followed by emotional intelligence and optimism. These findings expand the existing literature on the role of personal resources in mental health and highlight the need for their development in youths to help them cope more effectively and function better after being cyberbullied.

## 1. Introduction

Cyberbullying is recognised as a serious psychosocial problem that is prevalent in schools around the world [1,2]. Cyberbullying is a damaging and unwanted type of aggressive behaviour conducted using modern information and communication technology. It refers to repeated, aggressive and intentional acts, characterized by an imbalance of power between the victim and aggressor and where the victim has difficulty defending his or herself [3,4]. There are others term related to misbehaviour in cyberspace, such as cyber incivility and cyber harassment, but in this study we focus on the above cyberbullying definition. All these online aggressive behaviours offer perpetrators a number of advantages over face-to-face bullying methods: an infinite audience, anonymity, and no physical contact in harming others, to name a few. Reports of violence in the digital environment suggest that one in five young Spanish people aged 11–18 years have engaged in cyberbullying [5].

Some students are cyberbullied over a long period of time; this can have both short- and long-term consequences [6,7,8]. A growing body of research suggests an association between adolescents being cybervictimised and depressive symptoms. Both cross-sectional [8] and longitudinal [9] studies have found that depression is frequently identified as a negative outcome of cyberbullying victimisation. In other words, cybervictims appear to be more prone to suffering from psychological disorders, such as depression [9].

### 1.1. Personal Resources as Predictors of Depression Symptoms

When cybervictimisation is not managed appropriately, it is more likely that adolescent victims will develop internalising problems, such as depressive symptoms. However, evidence also suggests that there is variability in the person’s emotional reaction to being victimised—i.e., they do not always experience similar negative outcomes nor exhibit them to the same degree [10]. Moreover, while there has been a plethora of research on cyberbullying and internalising problems [11,12], there remain gaps in our knowledge regarding the role that personal factors play in determining the extent to which victimised adolescents may suffer with depressive symptoms. According to the basic ideas of the Diathesis–Stress Model [13], individuals with different positive personal resources can be differently influenced by similar stressful or negative events. This theory states that interactions between the individual and the cyberbullying context could explain the occurrence and development of depression [14]. Importantly, there are some well-known factors that, when present, have consistently reduced the association between depression and cyberbullying: emotional intelligence [7,8], gratitude [15], forgiveness [16,17,18] and optimism [19].

#### 1.1.1. Emotional Intelligence

Over the last two decades, evidence has accumulated that several factors can potentially act as predictors of adverse outcomes pursuant to cyberbullying victimisation. One of these is emotional intelligence (EI). EI may play an important role in promoting personal growth and positive relationships and health [20,21]. From the ability perspective, EI is defined as a group of skills related to perceiving, accessing, and generating emotions to assist with thought, the understanding and regulation of emotions and the development of emotional knowledge; as such, EI can promote emotional and intellectual growth [22]. Several studies have found that people (particularly adolescents), with high EI are more able to manage their emotions and others’ negative emotions and thereby are more able to improve their psychological wellbeing and prevent psychological maladjustment [8,23]. These results have been attributed to this high EI group’s superior affective regulatory processes—which, in turn, reduce the probability of negative mood states and emotional problems, such as those associated with mental disorders [23,24]. It thus seems fruitful to analyse whether the scarcity of emotional abilities can make adolescents susceptible to internalising problems, and thus developing depressive symptoms in the cyberbullying context.

#### 1.1.2. Gratitude

Another personal factor that could predict a youth’s response to online aggression is gratitude. Emmons and McCullough [25] defined grateful disposition as a generalised tendency to recognise that one has experienced a positive outcome—intentionally provided by another person or moral agent—and respond to that result with positive emotion. The gratitude is considered to be a life orientation in which the person notices and appreciates the good things that happen to them and when they express thanks to those responsible [26]. Previous studies have shown links between gratitude and lower levels of psychopathological symptoms; in particular, depression [27,28,29]. Furthermore, adolescents who report grateful moods also report greater subjective wellbeing, more optimism, and more social support; they also display more prosocial behaviours [30,31]. Being grateful renders individuals more prone to showing kindness, comprehension, support, and compassion toward themselves when negative vital events occur [28]. In the context of cyberbullying, a recent study has shown that grateful people who are aware of the positive things in life are less cybervictimised [32]. It could, thus, be worthwhile to investigate whether, when adolescents with a grateful attitude are cybervictimised, they develop less emotionally-related symptoms than their peers, displaying less gratitude. If these results were obtained, we could suggest that a posture of gratitude may be a crucial personal resource for adolescents navigating in cyberspace.

#### 1.1.3. Forgiveness

As with gratitude, forgiveness is considered a resource that may alleviate the negative outcomes of being bullied. Forgiveness may help bring an end to a cycle of violence in school and may promote a harmonious school culture [33,34]. Forgiveness is defined as a reduction in negative emotions (e.g., resentment, bitterness, anger, fear, or hostility) together with a change from negative to positive feelings, cognitions, and behaviours in relation to the perpetrator of an offence, that may include oneself, others, and God. [35]. Scholars have pointed out that adolescents who report higher forgiveness display better mental health [17,36]. Some empirical studies have specifically examined the relationship between forgiveness and cyberbullying [37] and have underscored the importance of forgiveness in cyberbullying contexts and related outcomes. A recent systematic review showed that adolescents who are cyberbullied, but report being able to forgive, tend to report lower levels of mental health difficulty than victims who are unable to forgive [38]. In total, then, forgiveness appears to be a key element for helping victims overcome interpersonal transgressions and improving general wellbeing [39].

#### 1.1.4. Optimism

Another positive psychological construct that has consistently been found to be associated with psychological adjustment is optimism [40,41]. Dispositional optimism is the generalised expectation that good things will happen in the future [14,42]. Research has shown that optimism is associated with greater vital satisfaction and psychological wellbeing [43] and with better interpersonal conflict management [44], and is negatively associated with depression, hopelessness, and suicidal ideation [45]. Likewise, some studies have suggested that optimism and pessimism may be associated with being victimised or targeted by bullies [46]. Niu et al. [14] found that optimists who suffer a specific type of cyberbullying (e.g., to be ignored and excluded by others) suffer less depression than pessimists, possibly because optimists look for some positive interpretations when coping with negative events; they also may use more positive coping strategies. In summary, the scientific literature suggests that optimism could be a personal resource that contributes to the development of psychological well-being and conversely predicts the development of potential risks of negative or stressful experiences, such as being victimised by electronic devices.

### 1.2. Rationale for this Study

Despite the association between victimisation and depressive symptoms, little attention has been paid to the literature to examine which positive personal resources may mitigate the effects of cybervictimisation on adolescents’ adjustment.

Hence, the purpose of this study was twofold. The first aim was to examine potential significant differences between the personal resources of two groups of victims of cyberbullying: those with depressive symptomatology and the non-depressive symptomatology group. We hypothesised that cybervictims with symptoms of depression would report lower scores on different personal resources, as most of the previous research in this area has shown that personal resources are negatively related to psychopathology [8,14,28,37]. Second, as far as we know, no previous study has examined the independent and joint contributions of several personal resources to clinical symptoms of being cybervictimised. Hence, a further objective was to explore the role of specific personal resources as predictors of depressive symptoms in the context of cyberbullying; we expected that all personal resource constructs would contribute to variance in symptoms of depression.

## 2. Materials and Methods

### 2.1. Participants and Procedures

A convenience sample of adolescents from six high schools in Málaga Province (Andalusia; Spain) participated in this cross-sectional study (N = 1622). Their ages ranged from 12 to 17 years (M = 14.03, SD = 1.47). A subsample of those who were victims of cyberbullying was selected according to the criteria used by Elipe, De-la-Oliva, and Del Rey [47]—i.e., those who reported that they were subjected to at least one of the stated cyberbullying behaviours at least “once or twice a month”. Thus, the final sample comprised 251 adolescents (50.2% girls) with ages ranging from 12–17 years. The distribution of academic level was as follows: 4% were attending classes of the first course of compulsory secondary education; 12.4% were attending classes of the second course of compulsory secondary education; 33.9% were attending the third course; 26.7% were attending the fourth course; the remainder (23.1%) were attending classes at the A level of post-compulsory education. This sample was divided into two groups based on scores on the Child Depression Inventory Short [48]; those with scores ≥ 9 were assigned to the depression group and the rest to the non-depression group (the two subsamples are described in more detail below). This study was approved by the Ethics Committees of the University of Málaga (62-2016- H).

#### 2.1.1. Depression Group of Cybervictims

This group comprised 89 participants (62.9% male) ranging in age from 13 to 17 years (M = 14.87, SD = 1.08). Regarding grades, 1.1% of the students were attending the first course of compulsory education; 7.9% were attending the second course of compulsory education; 47.2% were attending the third course; 25.8% the fourth course; 18% were attending classes at the A level of post-compulsory education.

#### 2.1.2. Non-Depression Group of Cybervictims

This group was comprised of 162 adolescents (42.6% male) ranging in age from 12 to 17 years, with a mean age of 15.14 years (SD = 1.43); 5.6% were attending the first course of compulsory education; 14.8% were attending the second course of secondary education; 26.5% were attending the third course, 27.2% the fourth course; 25.9% were attending classes at the A level of post-compulsory education.

### 2.2. Measures

The European Cyberbullying Intervention Project Questionnaire (ECIPQ; [49,50]) consists of 22 items assessing the frequency of performing cyberbullying behaviours. We used the subscale of cyberbullying victimisation; this scale comprises 11 items on which respondents indicate how frequently they have experienced each form of cyberbullying in the last two months using a five-point Likert scale where 0 = never; 1 = yes, once or twice; 2 = yes, once or twice a month; 3 = yes, about once a week and 4 = yes, more than once a week (for example, “Someone has said nasty things to me or has insulted me via email or SMS”; “Someone has threatened me through messages on the internet or SMS”). This instrument demonstrated good psychometric properties [50]. In our sample, Cronbach’s alpha was adequate (α = 0.75). As mentioned, we used the criterion proposed by Elipe and colleagues [47] to classify some participants as “non-victims” (those who marked the “never” or “yes, once or twice” option in all items ) and some as “victims” (those who indicated that one of the behaviours happened to them “once or twice a month” or “once or twice a week” or more).

The Children’s Depression Inventory-Short (CDI-S; [48,51]) consists of 10 items and is a shorter version of the Children’s Depression Inventory (27 items; [51]). The CDI-S captures the key symptoms of depression among adolescents. There are three response options for each item (with scores of 0 to 2); item scores are summed to yield a total score ranging from 0 (not depressed) to 20 (very high risk of depression). This measure was used to determine whether participants reported a below average, average, or above average level of depressive symptoms. We used the criterion proposed by Yu and colleagues [52] to assign participants to the non-depressed group (scores < 9) or the depressed group (scores ≥ 9). The scale demonstrated good psychometric properties [48]; in our sample, Cronbach’s alpha was 0.76.

The Wong and Law Emotional Intelligence Scale (WLEIS; [53]) consists of 16 items organised into four-item subscales: emotional self-appraisal (SEA), others-emotion appraisal (OEA), regulation of emotion (ROE), and use of emotion (UOE). Responses are given using a seven-point Likert scale ranging from 0 (totally disagree) to 6 (totally agree). As we were interested in the overall construct, we summed the subscale scores to yield a global perceived EI score; higher scores indicate greater EI. Previous studies have demonstrated the validity and reliability of the Spanish version of the WLEIS in adolescents [54]. In the present study, Cronbach’s alpha for the scale was 0.87.

The Gratitude Questionnaire (GQ; [55,56]) is a five-item measure of grateful disposition to which responses are given using a seven-point Likert scale ranging from 1 (strongly disagree) to 7 (strongly agree). GQ scores have demonstrated satisfactory psychometric properties [55]. We used the Spanish adaptation of the GQ [56], which had a Cronbach’s alpha of 0.80 in our sample.

The Revised Life Orientation Test (LOT-R; [57]) is a six-item (there are additional 4 filler items) measure of individual differences in dispositional optimism and pessimism. We used only the optimism subscale; higher scores reflect a greater tendency to expect positive outcomes. We used a well-validated Spanish version of the instrument [58]. There is extensive evidence on the reliability and validity of the LOT-R and its subscales [42]; however, some studies have reported low reliability indices (0.53 and 0.64 for the optimism and pessimism subscales, respectively) when analysing the two subscales separately [59]. In this study, the Cronbach’s alpha for the optimism subscale was 0.52.

The Brief Multi-Dimensional Measure of Religiousness and Spirituality (BMMRS; [60]) was used to assess dimensions of dispositional forgiveness. Three single-item measures of forgiveness were used: forgiveness of self (e.g., “I have forgiven myself for things that I have done wrong”), forgiveness of others (e.g., “I have forgiven those who hurt me”), and forgiven by God (e.g., “I know that God forgives me”). Responses were given using a four-point Likert scale ranging from 1 (never) to 4 (almost always). These items were previously used to assess distinct dimensions of forgiveness and as a broad measure of forgiveness [61]; they exhibited adequate internal consistency and good test–retest reliability [62].

### 2.3. Data Analysis

Statistical analyses were conducted using SPSS version 24.0 (IBM Corporation, Armonk, NY, USA). First, we computed pairwise Pearson’s correlations between personal resources separately for each group in order to assess associations between the study variables. Then, we examined group differences in personal resources using MANOVA. Finally, logistic regression was used to determine which personal resources were the most important predictors of level of depression symptoms. We computed a series of stepwise logistic regression models. In the first analysis, we examined the effect of the control variables (sex and age) in the two groups; in the subsequent analyses, we added the personal resource variables as independent variables. To detect any multicollinearity between different personal resource variables, we used the variance inflation factor (VIF), with values greater than 10 considered to represent a multicollinearity problem.

## 3. Results

### 3.1. Correlations between Personal Resources

In the depression group, the significant correlations between the personal resource variables ranged from 0.33 (*p* < 0.01; optimism and forgiven by God) to 0.45 (*p* < 0.01; gratitude) whereas, in the non-depression group, they ranged from 0.20 (*p* < 0.01; optimism) to 0.30 (*p* < 0.01; EI). Using Cohen’s criteria [63] these results indicate moderate to strong correlations between the variables (Table 1) and suggest that multivariate analyses should be performed to examine the unique relationships between personal resources and group membership (while still accounting for the mutual correlations).

### 3.2. Group Differences in Personal Resources

We calculated descriptive statistics (mean and standard deviation) for all personal resource variables for both groups. The results are shown in Table 2.

We used MANOVA to assess whether there was an overall multivariate group difference in reported personal resources for the separate group of victimised adolescents with depressive and with non-depressive symptomatology. Sex was also included as independent variable in order to examine potential main and interaction effects. The results showed there was no main effect of sex (Wilks’s λ = 0.99; *F* (4244) = 0.45; *p* = 0.76) and no group by sex interaction (Wilks’s λ = 0.99; *F* (4244) = 2.02; *p* = 0.09). There was, however, a group difference in the depressive symptomatology and non-depressive symptomatology samples (Wilks’s λ = 0.66; *F* (4244) = 30.71; *p* = 0.000). Post hoc *t*-tests (Table 2) showed that the non-depression group reported higher levels of EI, gratitude, optimism and forgiveness (of self of others).

### 3.3. Prediction of Depression Group Membership: Logistic Regression Analysis

We performed logistic regression to determine which of the different personal resource variables best distinguished the two groups, using group membership as a binary dependent variable and the personal resource variables as independent variables. Because sex and age commonly generate significant differences, they were included as covariates. Sex variable was coded as 1 = male and 2 = female.

In the first step, sex and age were entered as independent variables, yielding a significant model (chi^2^ (2) = 12.79; *p* = 0.002), which explained 5% of the variance in group membership (Cox and Snell’s *R*^2^). Next, the personal resources were added as independent variables, which increased the explained variance by 35.2%. The total variance explained was 40.2% (chi^2^ (8) = 108.71, *p* = 0.000). Table 3 presents the results of this logistic regression, with sex, age, and the five personal resources as independent variables. The Wald statistic was used to determine the significance of the contribution of the independent variables. The standardised logistic regression coefficient (standardised *B*) was used to measure the relative influence of the different independent variables. Finally, the VIF was used to examine the multicollinearity between variables.

Table 3 shows that, after controlling for sex and age, several personal resources showed to be independent predictors of depressive symptomatology group membership. Gratitude was the best predictor of group membership, with low gratitude predicting membership of the depression group. VIF ranged from 1.00 to 1.49, indicating an absence of multicollinearity between different predictors.

The model presented in Table 3 includes several variables that were not predictors of group membership; these may have artificially increased the percentage of variance explained by the model. Therefore, we calculated a second model in which the significant predictors were the sole variables included (Table 4). VIF ranged from 1.00 to 1.47, indicating an absence of multicollinearity between different predictors. This final model, which included emotional intelligence, gratitude, optimism, and forgiveness of others, explained 37.4% of the variance in group membership (chi^2^(5) = 105.79, *p <* 0.001).

## 4. Discussion

This study examined the relationships between personal resources and depressive symptoms in adolescent victims of cyberbullying, comparing depressive symptomatology and non-depressive symptomatology groups. We hypothesised that members of the depression group would report lower scores in all examined personal resources. In addition, we explored the separate and joint contributions of various personal resources to variance in symptoms of depression in cyberbullying victims. We predicted that the personal resources examined would contribute to variance in depressive symptoms.

With regard to the first goal of this study, we found that the group with a high level of depressive symptoms had lower scores in several personal resources, which is in line with earlier research [17,64]. The depression group reported lower scores on EI, gratitude, optimism, and forgiveness of self than the non-depression group. The findings regarding EI are consistent with previous research and suggest that individuals who are better at perceiving, understanding and managing their emotions and who better understand the implications of their emotional states are likely to cope more successfully with negative experiences (e.g., being cyberbullied), because they can use more effective emotion regulation processes [65]. It has, in fact, been shown that emotional abilities reduce negative mood states associated with psychopathology [66].

Regarding gratitude, our findings show a robust association between this factor and the non-depressive symptomatology group. This result is consistent with previous studies confirming a negative link between gratitude and the presence of several psychopathological outcomes [27,28]. Following the Broaden-and-Build Theory [67], positive emotions appear to enlarge the cognitive context and build enduring personal resources [68]. Fredrickson [69] states that people who experience positive emotions broaden their cognitive and behavioural repertoires, widening the array of thoughts and actions that come to mind. Besides, the Broaden-and-Build Theory establishes that, if negative emotions (e.g., negative emotions that commonly appear after cybervictimisation) narrow the momentary thought–action repertoire, positive emotions broaden that same repertoire too. This implies that positive emotions should have an undoing effect on the lingering consequences of negative emotions [70,71] enabling people to become more resilient. In the cyberbullying context, it is tentative to think that cybervictims who experience positive emotions, such as gratitude, could transform themselves, becoming more resilient against peer aggression than their counterparts with low gratitude and, therefore, not develop depressive symptoms. Accordingly, Petrocchi and Couyoumdjian [28] found that grateful people may possess a world view that is more focused on appreciating the good things in life and, further, showing less self-criticism and self-attacking when facing life circumstances.

Concerning optimism, our findings are consistent with previous research, showing a negative association between dispositional optimism and depression. Moreover, optimistic adolescents have been shown to be more likely to have successful peer relationships and to experience less depression [72,73,74]. One plausible reason is that optimists use different mechanisms for coping with a stressful event such as cybervictimisation [75]. For example, optimistic individuals use acceptance as a coping strategy, whereas pessimist people make greater use of overt denial. Acceptance does not mean giving up; rather, it involves a restructuring of one’s perception and goals and may actually serve to keep a person focused on those goals and engaged in life [76].

With regard to forgiveness, cyberbullying is considered an interpersonal transgression; research has shown that experiences of being bullied or cyberbullied are associated with an increased risk of stressful reactions, negative emotions, and vengeful motivations [38]. Forgiveness attenuates the stress reaction and its negative consequences on psychological and physical health [77]. In this regard, our results are mixed. On the one hand, the findings of this study indicate a negative and significant association between forgiveness of self and depressive symptomatology, in line with past studies [17,78]. On the other hand, although most literature highlights the benefits of forgiveness in different samples [38], our results show that, in the cyberbullying context, forgiveness of others is related to the development of depressive symptomatology. One possible explanation might be that forgiveness of others facilitates coping with such offenses [33] via regulation of one’s own negative emotions, which yields a measure of control over the situation [79]. Likewise, people coped more effectively when they perceived that they had greater control over their circumstances [80]. However, when adolescents are bullied in cyberspaces, they can feel that they have no control of the situation [81]. According to some authors, after adolescents experience an offense difficult to eliminate directly or change in some degree, they often use forgiveness as a coping strategy focused on emotions [38]. However, adolescents’ understanding of forgiveness might affect the health implications of cyberbullying transgressions. Some research has found negative relationships between benevolent motivations and psychological adjustment [82,83]. According to our results, it is possible that forgiving offenders in the online context might be a negative coping strategy, more associated with negative outcomes in adolescent mental health.

Regarding to the second goal, the present study examined the joint and separate contribution of different personal resources to variance in symptoms of depression. We found that, after controlling for sex and age, all personal resources contributed independently to the prediction of depression group membership. As expected, the set of personal resources measured jointly explained a considerable amount of the variance in group membership. These findings support the idea that these personal resources involve somewhat similar emotional and cognitive processes sharing some conceptual overlap. Nonetheless, it is worth noting the finding regarding their independent contributions to depression. According to the results, it seems clear that the strongest predictor of depression level is gratitude (followed by emotional intelligence, optimism, and forgiveness of others in this order). The link between gratitude and wellbeing is well-known. There is consistent evidence that gratitude predicts wellbeing and social variables [55,84,85]. Given that dispositional gratitude is an orientation to the positive in the world, which can be contrasted with the depressed person’s tendency to focus on the negative in the self, world, and future [86], we suggest that having high levels of gratitude protects against depression.

Gratitude not only appeared as an important conversely predictor of depressive symptomatology as the results of our study suggest that depression is predicted by other resources. In this study, emotional abilities (or lack thereof) were found to be a good predictor of depressive symptomatology. This finding confirms those of other studies that suggest adolescents with high EI are more able to manage their emotions and others’ negative emotions for improving psychological wellbeing and preventing psychological maladjustment [8,23]. Besides, this study suggests that the kind of aggression (online or face-to-face) is important. Similarly, some research has found that it is not always favourable to forgive the offense [82,83,87]. So, it is worth noting that cyberspace presents a bad context for forgiving, because people cope more effectively with offenses when they feel they have greater control over their circumstances [80]. Finally, individuals who are optimistic have been shown to have better psychological adjustment (e.g., emotional wellbeing, adaptive coping) than those who are less optimistic [88]. It looks like optimistic people would look forward to a positive outcome and seek out some positive interpretations when faced with negative situations [14]. Our results confirm previous studies, suggesting that optimism is a negative predictor of depressive symptoms [89].

### Theoretical and Practical Implications

From the positive approach of personal resources, our results suggest that the appearance of depression in cyberbullying victims should not be traced to one specific personal resource but rather to the joint action of various resources. This has two important implications. One is that theoretical: studies on the relationship between personal resources and psychological adjustment should consider several resources simultaneously, rather than focusing on a single resource. This could provide further evidence on the conditions under which a resource may be more or less relevant to a particular psychological outcome. Along these lines, another practical implication is related to the use of comprehensive programmes for preventing symptoms of depression among victimised adolescents as well as using screening evaluations to detect risk factors. Given that cyberbullying is difficult to eradicate, risk factors should not be the sole focus; interventions to cultivate and strengthen protective factors in the adolescent victims who have low levels of these resources should also be introduced in order to prevent the negative outcomes on the mental health. This would allow for better coping with negative life situations [90].

Although our findings provide promising empirical evidence that personal resources protect against the development of psychopathology, some limitations should be acknowledged. The use of a cross-sectional design prevents us from drawing conclusions about the direction of influence. It should also be noted that we used a self-report measure of symptoms of depression. Future studies should use both self-reports and other forms of data, such as interviews or expert judgements. It might be worth using diaries to investigate whether within-person changes in personal resources are related to changes in functioning. Although a multi-dimensional approach to assessment of forgiveness was used, the use of single-item measures may represent a limitation. Future studies should incorporate more comprehensive assessments of forgiveness that consider situational factors. Nevertheless, it is worth pointing out that Worthington and colleagues [91] have suggested that dispositional forgiveness is positively associated with health; in addition, previous research—including factor analyses comparing single- and multiple-item indicators—has suggested that they are similarly sensitive [92].

These limitations notwithstanding, our research suggests that certain personal resources are closely related to reported symptoms of depression. A strength of the study is that it compared groups with and without depressive symptoms. Our investigation also provides some new insight and guidance for the development of interventions based on positive psychology [93] that are designed to promote the development of strengths that protect against the psychological outcomes of being cyberbullied, including depression.

## 5. Conclusions

As far as we know, our study is the first to assess the associations between various personal resources and depressive symptoms in a sample of adolescent victims of cyberbullying. Our results contribute to the consistent body of literature, indicating that personal resources have beneficial effects on health and functioning [93,94]. Although prospective research is needed to confirm this, gratitude, EI, optimism, and forgiveness may be negatively associated with psychopathology. This implies that enhancing personal resources would help to reduce the psychological distress and psychopathology that often precipitate severe negative outcomes, such as suicide, amongst adolescents [95].

## Figures and Tables

**Table 1:** Person intercorrelations between positive personal resources for non-depressive symptomatology group (*n* = 162, above diagonal) and depressive symptomatology group (*n* = 89, below diagonal).

Personal Resources	1	2	3	4	5	6
1. Emotional Intelligence	-	0.30 **	0.20 **	0.21 **	0.01	0.05
2. Gratitude	0.45 **	-	0.26 **	0.10	0.22 **	0.08
3. Optimism	0.33 **	0.38 **	-	0.07	0.04	0.09
4. Forgiveness of self	0.35 **	0.24 *	0.44 **	-	−0.02	0.05
5. Forgiveness of others	−0.01	0.05	−0.01	0.16	-	0.03
6. Forgiven by God	0.33 **	0.40 **	0.21 *	0.22 *	−0.21 *	-

Note. * *p* < 0.05; ** *p* < 0.01.

**Table 2:** Differences in positive personal resources scores between depressive symptomatology and non-depressive symptomatology group: means, standard deviations and *t*-test.

Positive Personal Resources	Non-Depressive Symptomatology Group (*n* = 162)		Depressive Symptomatology Group (*n* = 89)		*t*-Test for Equality of Means	
	**M**	**SD**	**M**	**SD**	***t***	***p***
Emotional Intelligence	4.93	0.83	3.96	1.07	7.93	0.00
Gratitude	5.59	1.02	4.28	1.15	9.21	0.00
Optimism	11.24	2.21	8.92	2.34	7.45	0.00
Forgiveness of self	2.49	0.88	2.05	0.89	3.72	0.00
Forgiveness of others	2.65	0.88	2.92	0.86	−2.29	0.22
Forgiven by God	2.35	1.17	2.06	1.12	1.89	0.59

**Table 3:** Identification of positive personal resources distinguishing depressive symptomatology (*n* = 89) and non-depressive symptomatology (*n* = 162) group membership: logistic regression analysis.

Predictors	B	SE *B*	Wald	*p*	Odds Ratio	VIF
Sex	−0.72	0.34	4.49	0.03	0.48	1.00
Age	−0.17	0.13	1.89	0.16	0.83	1.02
Emotional Intelligence	−0.51	0.19	7.29	0.00	0.59	1.49
Gratitude	−0.80	0.17	20.69	0.00	0.44	1.42
Optimism	−0.20	0.07	6.66	0.01	0.81	1.33
Forgiveness of self	−0.12	0.20	0.40	0.52	0.88	1.11
Forgiveness of others	0.48	0.19	5.86	0.01	1.61	1.03
Forgiven by God	0.15	0.15	0.96	0.32	1.16	1.06

Note. Total explained variance (Cox and Snell R^2^) = 40.2%; Significance model = chi^2^(9) = 325.62; *p* < 0.001.

**Table 4:** Distinction between depressive symptomatology (*n* = 89) and non-depressive symptomatology (*n* = 162) group membership: final logistic regression analysis.

Predictors	B	SE *B*	Wald	*p*	Odds Ratio	VIF
Sex	−0.67	0.33	3.97	−0.08	0.04	1.00
Emotional Intelligence	−0.54	0.18	8.73	0.00	0.00	1.47
Gratitude	−0.75	0.17	19.37	0.00	0.47	1.38
Optimism	−0.22	0.07	8.28	0.00	0.80	1.30
Forgiveness of others	0.44	0.19	5.26	0.02	1.55	1.02

Note. Total explained variance (Cox and Snell R^2^) = 37.4%; Significance model = chi^2^(5) = 105.79; *p* < 0.001.

## References

[1] Kowalski R.M., Giumetti G.W., Schroeder A.N., Lattanner M.R. (2014). Bullying in the digital age: A critical review and meta-analysis of cyberbullying research among youth. Psychol. Bull..

[2] Zych I., Ortega-Ruiz R., Marín-López I. (2016). Cyberbullying: A systematic review of research, its prevalence and assessment issues in Spanish studies. Psicol. Educ..

[3] Berne S., Frisén A., Oskarsson J. (2020). High school students’ suggestions for supporting younger pupils counteract cyberbullying. Scand. J. Psychol..

[4] Smith P.K., Mahdavi J., Carvalho M., Fisher S., Russell S., Tippett N. (2008). Cyberbullying: Its nature and impact in secondary school pupils. J. Child Psychol. Psychiatry.

[5] Calmaestra J., Rodríguez-Hidalgo A.J., Mero-Delgado O., Solera E. (2020). Cyberbullying in Adolescents from Ecuador and Spain: Prevalence and Differences in Gender, School Year and Ethnic-Cultural Background. Sustainability.

[6] Cañas E., Estévez E., Marzo J.C., Piqueras J.A. (2019). Psychological adjustment in cybervictims and cyberbullies in secondary education. Ann. Psychol..

[7] Extremera N., Quintana-Orts C., Mérida-López S., Rey L. (2018). Cyberbullying Victimization, Self-Esteem and Suicidal Ideation in Adolescence: Does Emotional Intelligence Play a Buffering Role?. Front. Psychol..

[8] Estévez E., Estévez J.F., Segura L., Suárez C. (2019). The Influence of Bullying and Cyberbullying in the Psychological Adjustment of Victims and Aggressors in Adolescence. Int. J. Environ. Res. Public Health.

[9] Camerini A.L., Marciano L., Carrara A., Schulz P.J. (2020). Cyberbullying perpetration and victimization among children and adolescents: A systematic review of longitudinal studies. Telemat. Inform..

[10] Hinduja S., Patchin J.W. (2017). Cultivating youth resilience to prevent bullying and cyberbullying victimization. Child Abuse Negl..

[11] Gini G., Card N.A., Pozzoli T. (2018). A meta-analysis of the differential relations of traditional and cyber-victimization with internalizing problems. Aggress. Behav..

[12] Kowalski R.M., Limber S.P., McCord A. (2019). A developmental approach to cyberbullying: Prevalence and protective factors. Aggress. Violent Behav..

[13] Monroe S.M., Simons A.D. (1991). Diathesis-stress theories in the context of life stress research: Implication for the depressive disorders. Psychol. Bull..

[14] Niu G.-F., Zhou Z.-K., Sun X., Yu F., Xie X.-C., Liu Q.-Q., Lian S.-L. (2018). Cyber-ostracism and its relation to depression among Chinese adolescents: The moderating role of optimism. Pers. Individ. Dif..

[15] Rey L., Quintana-Orts C., Mérida-López S., Extremera N. (2019). Being Bullied at School: Gratitude as Potential Protective Factor for Suicide Risk in Adolescents. Front. Psychol..

[16] Hirsch J.K., Webb J.R., Jeglic E.L. (2011). Forgiveness, Depression, and Suicidal Behavior Among a Diverse Sample of College Students. J. Clin. Psychol..

[17] Quintana-Orts C., Rey L. (2018). Forgiveness, Depression, and Suicidal Behavior in Adolescents: Gender Differences in this Relationship. J. Genet. Psychol..

[18] Barcaccia B., Pallini S., Baiocco R., Salvati M., Saliani A.M., Schneider B.H. (2018). Forgiveness and friendship protect adolescent victims of bullying from emotional maladjustment. Psicothema.

[19] Navarro R., Ruiz-Oliva R., Larrañaga E., Yubero S. (2015). The Impact of Cyberbullying and Social Bullying on Optimism, Global and School-Related Happiness and Life Satisfaction Among 10–12-year-old Schoolchildren. Appl. Res. Qual. Life.

[20] Mayer J.D., Roberts R.D., Barsade S.G. (2008). Human abilities: Emotional intelligence. Annu. Rev. Psychol..

[21] Sánchez-Álvarez N., Extremera N., Fernández-Berrocal P. (2016). The relation between emotional intelligence and subjective well-being: A meta-analytic investigation. J. Posit. Psychol..

[22] Mayer J.D., Caruso D.R., Salovey P. (2016). The Ability Model of Emotional Intelligence: Principles and Updates. Emot. Rev..

[23] Gomez-Baya D., Mendoza R., Paino S., de Matos M.G. (2017). Perceived emotional intelligence as a predictor of depressive symptoms during mid-adolescence: A two-year longitudinal study on gender differences. Pers. Individ. Differ..

[24] Martins A., Ramalho N., Morin E. (2010). A comprehensive meta-analysis of the relationship between Emotional Intelligence and health. Pers. Individ. Differ..

[25] Emmons R.A., McCullough M.E. (2003). Counting blessings versus burdens: An experimental investigation of gratitude and subjective well- being in daily life. J. Pers. Soc. Psychol..

[26] Wood A.M., Joseph S., Maltby J. (2009). Gratitude predicts psychological well-being above the big twelve facets. Pers. Individ. Differ..

[27] Emmons R.A., Stern R. (2013). Gratitude as a psychotherapeutic intervention. J. Clin. Psychol..

[28] Petrocchi N., Couyoumdjian A. (2016). The impact of gratitude on depression and anxiety: The mediating role of criticizing, attacking, and reassuring the self. Self Identity.

[29] Stoeckel M., Weissbrod C., Ahrens A. (2014). The adolescent response to parental illness: The influence of dispositional gratitude. J. Child Fam. Stud..

[30] Froh J.J., Bono G., Emmons R. (2010). Being grateful is beyond good manners: Gratitude and motivation to contribute to society among early adolescents. Motiv. Emot..

[31] Froh J.J., Yurkewicz C., Kashdan T.B. (2009). Gratitude and subjective well-being in early adolescence: Examining gender differences. J. Adolesc..

[32] Yudes C., Rey L., Extremera N. (2020). Predictive factors of cyberbullying perpetration amongst spanish adolescents. Int. J. Environ. Res. Public Health.

[33] Egan L.A., Todorov N. (2009). Forgiveness as a coping strategy to allow school students to deal with the effects of being bullied: Theoretical and empirical discussion. J. Soc. Clin. Psychol..

[34] Hui E.K., Tsang S.K., Law B. (2011). Combating school bullying through developmental guidance for positive youth development and promoting harmonious school culture. Sci. World J..

[35] Webb J.R., Toussaint L., Conway-Williams E. (2012). Forgiveness and health: Psycho-spiritual integration and the promotion of better healthcare. J. Health Care Chaplain..

[36] Janse Van Rensburg E., Raubenheimer J.E. (2015). Does forgiveness mediate the impact of school bullying on adolescent mental health?. J. Child Adolesc. Ment. Health.

[37] Quintana-Orts C., Rey L. (2018). Traditional bullying, cyberbullying and mental health in early adolescents: Forgiveness as a protective factor of peer victimisation. Int. J. Environ. Res. Public Health.

[38] Quintana-Orts C., Rey L., Worthington E.L. (2019). The Relationship Between Forgiveness, Bullying, and Cyberbullying in Adolescence: A Systematic Review. Trauma Violence Abus..

[39] Quintana-Orts C., Rey L. (2018). Forgiveness and cyberbullying in adolescence: Does willingness to forgive help minimize the risk of becoming a cyberbully?. Comput. Human Behav..

[40] Bailey T.C., Eng W., Frisch M.B., Snyder C.R. (2007). Hope and optimism as related to life satisfaction. J. Posit. Psychol..

[41] Hart S.L., Vella L., Mohr D.C. (2008). Relationships among depressive symptoms, benefit-finding, optimism, and positive affect in multiple sclerosis patients after psychotherapy for depression. Health Psychol..

[42] Scheier M.F., Carver C.S. (1985). Optimism, coping, and health: Assessment and implications of generalized outcome expectancies. Health Psychol..

[43] Daukantaitė D., Zukauskiene R. (2012). Optimism and subjective well-being: Affectivity plays a secondary role in the relationship between optimism and global life satisfaction in the middle-aged women. Longitudinal and cross-cultural findings. J. Happiness Stud..

[44] Srivastava S., McGonigal K.M., Richards J.M., Butler E.A., Gross J.J. (2006). Optimism in close relationships: How seeing things in a positive light makes them so. J. Pers. Soc. Psychol..

[45] Tucker R.P., Wingate L.R.R., O’Keefe V.M., Mills A.C., Rasmussen K., Davidson C.L., Grant D.M.M. (2013). Rumination and suicidal ideation: The moderating roles of hope and optimism. Pers. Individ. Differ..

[46] Rigby K. (2003). Consequences of bullying in schools. Can. J. Psychiatry.

[47] Elipe P., de la Oliva Muñoz M., Del Rey R. (2018). Homophobic Bullying and Cyberbullying: Study of a Silenced Problem. J. Homosex..

[48] Del Barrio M.V., Roa M.L., Olmedo M., Colodrón F. (2002). Primera adaptación del CDI-S a población española [First adaptation of the CDI-S for Spanish population]. Acción Psicológica.

[49] Del Rey R., Casas J.A., Ortega-Ruiz R., Schultze-Krumbholz A., Scheithauer H., Smith P.K., Thompson F., Barkoukis V., Tsorbatzoudis H., Brighi A. (2015). Structural validation and cross-cultural robustness of the European Cyberbullying Intervention Project Questionnaire. Comput. Hum. Behav..

[50] Ortega-Ruiz R., Del Rey R., Casas J.A. (2016). Evaluar el bullying y el cyberbullying validación española del EBIP-Q y del ECIP-Q [Assessing bullying and cyberbullying: Spanish validation of EBIPQ and ECIPQ]. Psicol. Educ..

[51] Kovacs M. (1992). Children’s Depression Inventory.

[52] Yu S., Clemens R., Yang H., Li X., Stanton B., Deveaux L., Lunn S., Cottrell L., Harris C. (2006). Youth and parental perceptions of parental monitoring and parent-adolescent communication, youth depression, and youth risk behaviors. Soc. Behav. Personal. Int. J..

[53] Wong C.-S., Law K.S. (2002). The effects of leader and follower emotional intelligence on performance and attitude: An exploratory study. Leadersh. Q..

[54] Extremera N., Rey L., Sánchez-Álvarez N. (2019). Validation of the Spanish version of the Wong Law Emotional Intelligence Scale (WLEIS-S). Psicothema.

[55] McCullough M.E., Emmons R.A., Tsang J.A. (2002). The Grateful Disposition: A Conceptual and Empirical Topography. J. Pers. Soc. Psychol..

[56] Rey L., Sánchez-Álvarez N., Extremera N. (2018). Spanish Gratitude Questionnaire: Psychometric properties in adolescents and relationships with negative and positive psychological outcomes. Pers. Individ. Differ..

[57] Scheier M.F., Carver C.S., Bridges M.W. (1994). Distinguishing optimism from neuroticism (and trait anxiety, self-mastery, and self- esteem): A reevaluation of the Life Orientation Test. J. Pers. Soc. Psychol..

[58] Ferrando P.J., Chico E., Tous J.M. (2002). Propiedades psicométricas del test de optimismo Life Orientation Test. Psicothema.

[59] Chang E.C., Sanna L.J., Yang K.-M. (2003). Optimism, pessimism, affectivity, and psychological adjustment in US and Korea: A test of a mediation model. Pers. Individ. Differ..

[60] Fetzer Institute/National Institute on Aging Working Group (2003). Multidimensional Measurement of Religiousness/Spirituality for Use in Health Research: A Report of the Fetzer Institute/National Institute on Aging Working Group, with Additional Psychometric Data.

[61] Knight J.R., Sherritt L., Harris S.K., Holder D.W., Kulig J., Shrier L.A., Chang G. (2007). Alcohol use and religiousness/spirituality among adolescents. South. Med. J..

[62] Harris S., Sherritt L., Holder D., Kulig J., Shrier L., Knight J. (2008). Reliability and validity of the Brief Multidimensional Measure of Religiousness/Spirituality among adolescents. J. Relig. Health.

[63] Cohen J. (1988). Statistical Power Analysis for the Behavioral Sciences.

[64] Salguero J.M., Extremera N., Fernández-Berrocal P. (2013). A meta-mood model of rumination and depression: Preliminary test in a non-clinical population. Scand. J. Psychol..

[65] Salovey P., Bedell B.T., Detweiler J.B., Mayer J.D., Snyder C.R. (1999). Coping intelligently: Emotional intelligence and the coping process. Coping: The Psychology of What Works.

[66] Hertel J., Schütz A., Lammers C. (2009). Emotional intelligence and mental disorder. J. Clin. Psychol..

[67] Fredrickson B.L. (2001). The role of positive emotions in positive psychology: The broaden-and-build theory of positive emotions. Am. Psychol..

[68] Fredrickson B.L. (1998). What good are positive emotions?. Rev. Gen. Psychol..

[69] Fredrickson B.L., Snyder C.R., Lopez S.J. (2002). Positive emotions. Handbook of Positive Psychology.

[70] Fredrickson B.L., Levenson R.W. (1998). Positive emotions speed recovery from the cardiovascular sequelae of negative emotions. Cogn. Emot..

[71] Fredrickson B.L., Mancuso R.A., Branigan C., Tugade M. (2000). The Undoing Effect of Positive Emotions. Motiv. Emot..

[72] Chang E.C. (1998). Dispositional optimism and primary and secondary appraisal of stressor: Controlling for confounding influences and relations to coping and psychological and physical adjustment. J. Pers. Soc. Psychol..

[73] Chang E.C. (1998). Does dispositional optimism moderate the relation between perceived stress and psychological well being? A preliminary investigation. Pers. Individ. Differ..

[74] Ridder D., Schreurs K., Bensing J. (2000). The relative benefits of being optimistic: Optimism as a coping resource in multiple sclerosis and Parkinson’s disease. Br. J. Health Psychol..

[75] Nes L.S., Segerstrom S.C. (2006). Dispositional optimism and coping: A meta-analytic review. Personal. Soc. Psychol. Rev..

[76] Carver C.S., Scheier M.F., Oettingen G., Sevincer A.T., Gollwitzer P. (2018). Generalized optimism. The Psychology of Thinking about the Future.

[77] Skaar N.R., Freedman S., Carlon A., Watson E. (2016). Integrating models of collaborative consultation and systems change to implement forgiveness-focused bullying interventions. J. Educ. Psychol. Consult..

[78] Toussaint L., Webb J.R., Worthington E.L. (2005). Theoretical and empirical connections between forgiveness, mental health, and well-being. Handbook of Forgiveness.

[79] van Oyen Witvliet C., Ludwig T.E., Vander Laan K.L. (2001). Granting forgiveness or harboring grudges: Implications for emotion, physiology, and health. Psychol. Sci..

[80] Hunter S.C., Boyle J.M.E. (2004). Appraisal and coping strategy use in victims of school bullying. Br. J. Educ. Psychol..

[81] Gaffney H., Farrington D.P., Espelage D.L., Ttofi M.M. (2019). Are cyberbullying intervention and prevention programs effective? A systematic and meta-analytical review. Aggress. Violent Behav..

[82] Barcaccia B., Howard B., Pallini S., Baiocco R. (2017). Bullying and the detrimental role of un-forgiveness in adolescents wellbeing. Psicothema.

[83] Ogurlu U., Sarıçam H. (2018). Bullying, forgiveness and submissive behaviors in gifted students. J. Child Fam. Stud..

[84] McCullough M.E., Tsang J.-A., Emmons R.A. (2004). Gratitude in intermediate affective terrain: Links of grateful moods to individual differences and daily emotional experience. J. Pers. Soc. Psychol..

[85] Wood A.M., Joseph S., Linley P.A. (2007). Coping style as a psychological resource of grateful people. J. Soc. Clin. Psychol..

[86] Beck A.T. (1976). Cognitive Therapy and the Emotional Disorders.

[87] Walters J.M., Kim-Spoon J. (2014). Are religiousness and forgiveness protective factors for adolescents experiencing peer victimization?. J. Aggress. Maltreat. Trauma.

[88] Carver C.S., Scheier M.F., Segerstrom S.C. (2010). Optimism. Clin. Psychol. Rev..

[89] Chang E.C., Martos T., Sallay V., Chang O.D., Wright K.M., Najarian A.S.M., Lee J. (2017). Examining optimism and hope as protective factors of suicide risk in Hungarian college students: Is risk highest among those lacking positive psychological protection?. Cognit. Ther. Res..

[90] Divecha D., Brackett M. (2020). Rethinking school-based bullying prevention through the lens of social and emotional learning: A bioecological perspective. Int. J. Bullying Prev..

[91] Worthington E.L., Berry J.W., Parrott L., Plante T.G., Sherman A.C. (2001). Unforgiveness, forgiveness, religion and health. Faith and Health: Psychological Perspectives.

[92] Dollinger S.J., Malmquist D. (2009). Reliability and validity of single-item self-reports: With special relevance to college students’ alcohol use, religiosity, study, and social life. J. Gen. Psychol..

[93] Seligman M.E.P., Steen T.A., Park N., Peterson C. (2005). Positive psychology progress: Empirical validation of interventions. Am. Psychol..

[94] Proctor C., Tsukayama E., Wood A.M., Maltby J., Eades J.F., Linley P.A. (2011). Strengths gym: The impact of a character strengths-based intervention on the life satisfaction and well-being of adolescents. J. Posit. Psychol..

[95] Zych I., Ortega-Ruiz R., Del Rey R. (2015). Systematic review of theoretical studies on bullying and cyberbullyingh: Facts, Knowledge, prevention and intervention. Aggress. Violent Behav..

